# Towards reproducible MRM based biomarker discovery using dried blood spots

**DOI:** 10.1038/srep45178

**Published:** 2017-03-27

**Authors:** Sureyya Ozcan, Jason D. Cooper, Santiago G. Lago, Diarmuid Kenny, Nitin Rustogi, Pawel Stocki, Sabine Bahn

**Affiliations:** 1 Department of Chemical Engineering and Biotechnology, University of Cambridge, Cambridge, United Kingdom; 2 Department of Chemical Engineering and Biotechnology, Psynova Neurotech Ltd, Cambridge, United Kingdom

## Abstract

There is an increasing interest in the use of dried blood spot (DBS) sampling and multiple reaction monitoring in proteomics. Although several groups have explored the utility of DBS by focusing on protein detection, the reproducibility of the approach and whether it can be used for biomarker discovery in high throughput studies is yet to be determined. We assessed the reproducibility of multiplexed targeted protein measurements in DBS compared to serum. Eighty-two medium to high abundance proteins were monitored in a number of technical and biological replicates. Importantly, as part of the data analysis, several statistical quality control approaches were evaluated to detect inaccurate transitions. After implementing statistical quality control measures, the median CV on the original scale for all detected peptides in DBS was 13.2% and in Serum 8.8%. We also found a strong correlation (*r* = 0.72) between relative peptide abundance measured in DBS and serum. The combination of minimally invasive sample collection with a highly specific and sensitive mass spectrometry (MS) technique allows for targeted quantification of multiple proteins in a single MS run. This approach has the potential to fundamentally change clinical proteomics and personalized medicine by facilitating large-scale studies.

Reproducible quantification of proteins across multiple samples is essential in biomarker research. Although immunoassays still remain the predominant method in clinical laboratories, they suffer from several drawbacks including batch to batch antibody variation, high cost and relatively large sample volume requirements^1^,^2^. Protein quantification using mass spectrometry (MS) overcomes some of these obstacles and offers sensitive, specific and reproducible data yielding fewer false positive and false negative detections. Consequently, over the last decade, MS has become increasingly important for quantitative proteomics^3^.

Quantification by MS can involve a targeted or an untargeted approach. Traditional hypothesis-free untargeted quantification uses a method commonly known as shotgun proteomics which aims to identify and quantify as many proteins as possible^4^,^5^. However, this approach often suffers from poor reproducibility, especially when analysing lower abundant proteins. On the other hand, the hypothesis-driven targeted approach, known as multiple reaction monitoring (MRM; also known as selective reaction monitoring), provides for sensitive and robust quantification of pre-selected proteins^6^,^7^. Both approaches have been widely used for quantitative analysis of proteins in various complex sample matrices including Dried Blood Spots (DBS) and serum^8^,^9^,^10^,^11^,^12^.

In MRM, a pre-selected specific peptide is fragmented by collision induced fragmentation and the intensity of the resulting fragment ions, called transitions, are measured. Stable isotope standard (SIS) peptides are commonly implemented as internal standards for MRM analyses using a technique known as stable isotope dilution^13^. The SIS peptides are synthetically produced with either a heavy arginine or lysine, and are added to the sample, ideally in a ratio of 1:1 to the endogenous peptides. As the SIS peptides have identical chromatographic, ionization and fragmentation properties as the endogenous peptides, they greatly improve the specificity of the acquired MRM data^14^. This is particularly important in large-scale clinical proteomics studies, where reproducibility is vital. The selection of peptides and interference-free transitions is also crucial for protein quantification. Various resources are available including PeptidePicker^15^, PeptideTracker^16^, SRMAtlas^17^,^18^, PeptideAtlas^18^, PASSEL^18^ and Passport Protein Assay Portal. These tools assist researchers by assembling publically available experimental data into databases. Such portals are very useful. However, both peptide and transition selections are affected by the biological matrix and experimental settings (digestion protocols, instrument specifications and instrument settings). Therefore, each study needs to be optimized in terms of achieving the highest sensitivity and specificity based on the biological samples and instrument specific settings.

Non-invasive diagnostics have an obvious appeal for both patients and clinicians, and are a focus of research in both academia and biopharma^19^,^20^. Several clinical trials of non-invasive diagnostic tools are underway worldwide for various diseases, bringing new concepts to the field of biomarker discovery, such as liquid biopsies^20^. For clinical proteomics, serum and plasma samples have been widely used as they are easier to obtain than other biological specimens, such as more invasive biopsies. However, the collection, shipment and storage of serum and plasma samples can be an obstacle, particularly, outside hospital environments. DBS is a form of sampling where blood samples are blotted and dried on a filter paper and offer an attractive and cost effective alternative as the approach is far less invasive and kits for self sample collection can be sent to the home. Samples can then be shipped and stored at room temperature until analysis. DBS sampling also reduces the infection risk as the blood samples are dried^21^. Furthermore, many analytes are more stable in a dried sample at room temperature than aqueous samples^22^,^23^. The use of DBS sampling in the clinical environment first came to prominence with the screening of new-borns for the metabolic disease phenylketonuria^24^ and is now utilized in screening for a wide range of other metabolic disorders. Although DBS sampling has predominantly been used in metabolite-based clinical diagnostics^25^,^26^, there is an emerging interest in the use of this alternative biological source in proteomics for biomarker discovery^8^,^9^,^10^,^27^,^28^; particularly for diseases such as psychiatric disorders in which patient recruitment is notoriously difficult and expensive. Despite the cost, collection, shipment and storage advantages, the complexity of DBS samples (including cellular components) represents a challenge for proteomics investigations. Consequently, further method development is required to achieve clinical utility as neither current serum nor plasma assays can be readily applied to DBS samples^12^. In this respect, targeted proteomic approaches provide an opportunity to overcome the limitations of DBS sample complexity.

Over the past decade, advances have been made in the use of DBS in proteomics, resulting in the successful identification of around one hundred proteins using targeted and untargeted MS methods^8^,^9^,^10^,^11^,^28^. However, in-depth research on the DBS proteome has been limited. The DBS proteome shows great similarity to the serum proteome but additionally contain proteins derived from red and white blood cells present in whole blood^8^,^28^. While previous studies have mainly focused on the feasibility of DBS sampling for profiling proteins in small sample sets, the practicality and reproducibility of large-scale DBS sampling is yet to be explored.

The aim of this study was to investigate whether we can reproducibly quantify targeted proteins in DBS samples. To this end, we selected 82 medium to high abundant proteins and evaluated: (1) existing statistical approaches for the identification of inaccurate peptide-transitions; (2) the reproducibility of relative peptide-transition abundances (including sample preparation and peptide detection) in DBS as compared to serum; and, (3) the correlation between relative peptide abundances measured in DBS and serum samples. In this particular application, the existing statistical approaches to detect inaccurate peptide-transitions all failed. Consequently, we developed an alternative approach.

## Results

**Figure 1:**
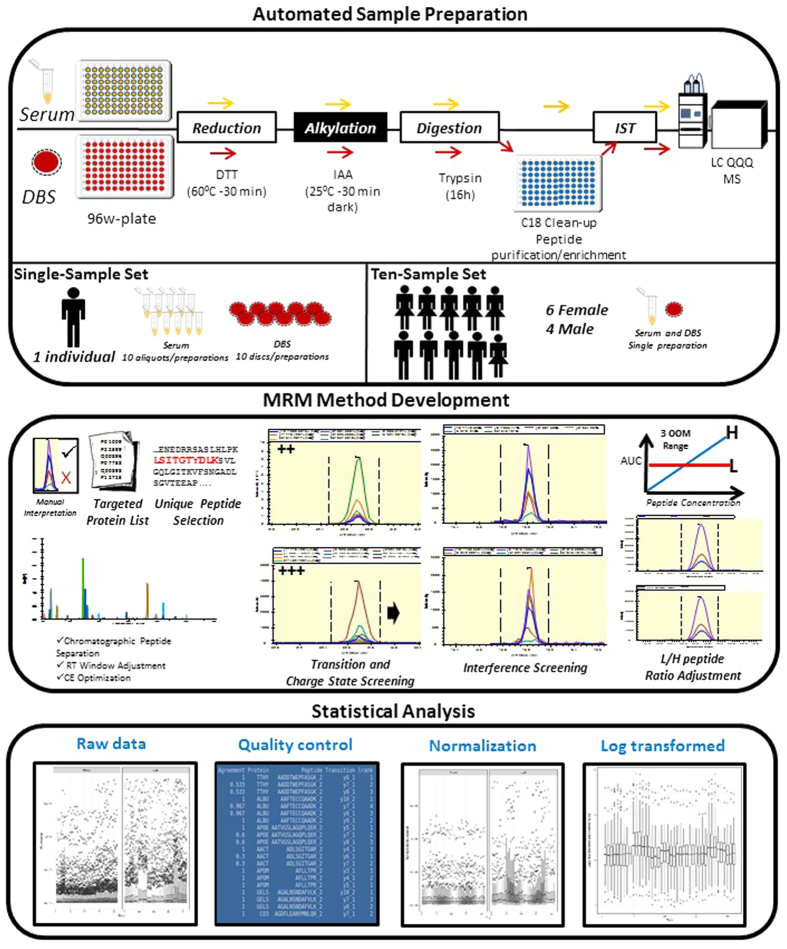
Detailed experimental workflow for targeted DBS protein quantification. The protocol consists of the following stages; (i) Automated sample preparation containing protein extraction and digestion, (ii) Multiplex MRM method development including protein/peptide selection, charge-state/transition filtering and endogenous/internal peptide ratio adjustment, (iii) Statistical analysis.

A comprehensive workflow including sample preparation, MRM multiplex assay development and statistical analysis is depicted in Fig. 1. DBS and serum samples were prepared in a 96-well plate format using an automated liquid handler to improve reproducibility. A conventional trypsin digestion approach was utilized for both the DBS and serum sample preparations. An additional solid phase extraction (SPE) step was implemented to the DBS workflow to reduce matrix complexity. Pooled DBS samples and Sigma serum (Human Sera S7023, Sigma Aldrich) were used as QC samples to optimize the experimental workflow as described in the materials and method sections.

### Identification of inaccurate peptide-transitions

The between run interference score^7^ which is based on the endogenous peptide only, by definition was detecting peptide-transitions with a correlation coefficient below the threshold level (*r* < 0.8) with the mean of their transition abundance for the corresponding peptide across the MS runs rather than inaccurate peptide-transitions. In addition, when several peptide-transitions were measured, an ‘outlying’ transition can affect the mean and result in only the outlying transition being retained. The score flagged (*r* < 0.8) 17.7% of DBS and 3.8% of serum peptide transitions as “inaccurate” (Table 1). However, the visual inspection revealed that almost all of the flagged transitions were erroneously flagged in the DBS and serum respectively (Table 1).

In the all-pairs approach^29^, 58.4% of DBS and 69.4% of serum peptide-transitions were flagged, mostly in error, as a result of the measurement error associated with the lower abundant peptide-transitions (Table 1). Given the problems with the measurement error of the lower abundant peptide-transitions as highlighted in the all-pairs approach, the simpler peptide-transition rank and proportion approaches, both based upon comparisons between the peptide and respective internal standard, also performed poorly, 24.4–34.3% and 51.5–62.5% respectively (Table 1). It was also observed that there is little consistency between the four approaches (Supplementary Table 1).

Given the poor performance of these four approaches when applied to our data for the identification of inaccurate peptide-transitions, we adopted an approach based on the most abundant peptide-transitions, which are the most reproducibly measured transitions (see Discussion). We selected the most abundant peptide-transitions with greater than 80% consistency across MS runs between endogenous and isotopically-labelled peptides and, for the most abundant peptide-transitions with less consistency, we visually checked the peptides for interference from the matrix and manually selected the most abundant transition based on the pooled plate QC samples. In addition, a relative abundance ratio filter was applied. Theoretically, the optimal ratio between endogenous and isotopically-labelled peptides is 1:1 on the original scale of measurement, but in practice, the complexity of optimization increases with the number of protein peptides. Consequently, we implemented the clinical guidelines suggesting a ratio range of 1:10 to 10:1^30^,^31^, importantly, calculated on the original scale of measurement.

### Coefficient of variation

**Figure 2:**
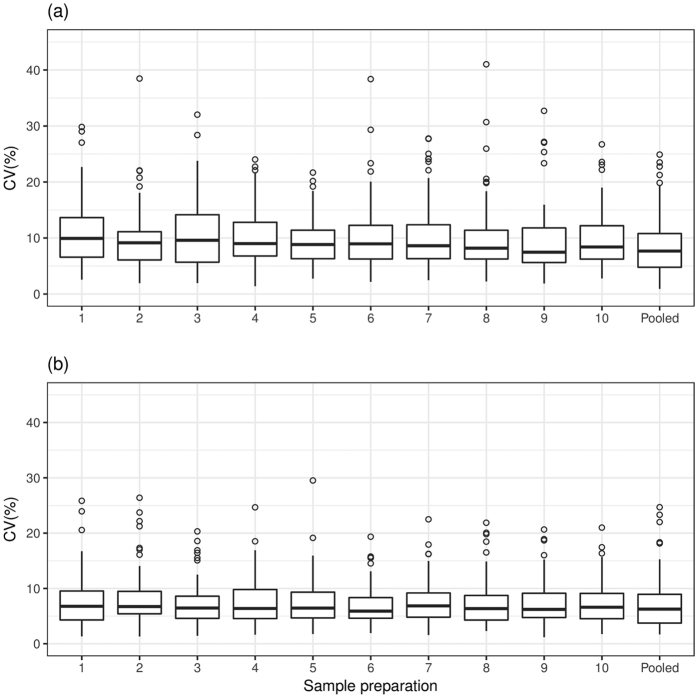
The ‘single-sample’ set CVs for 86 peptide-transitions (58 proteins) after abundance ratio filter exclusions applied, common to both DBS and serum. (**a**) The CV for peptide-transition abundance ratios measured in ten DBS sample preparations from the same healthy volunteer. (**b**) The CV for peptide-transition abundance ratios measured in ten serum sample preparations from reference Sigma Serum. Note that the CVs were based on eight injections for each sample preparation. Pool – pooled sample of all ten sample preparations.

**Figure 3:**
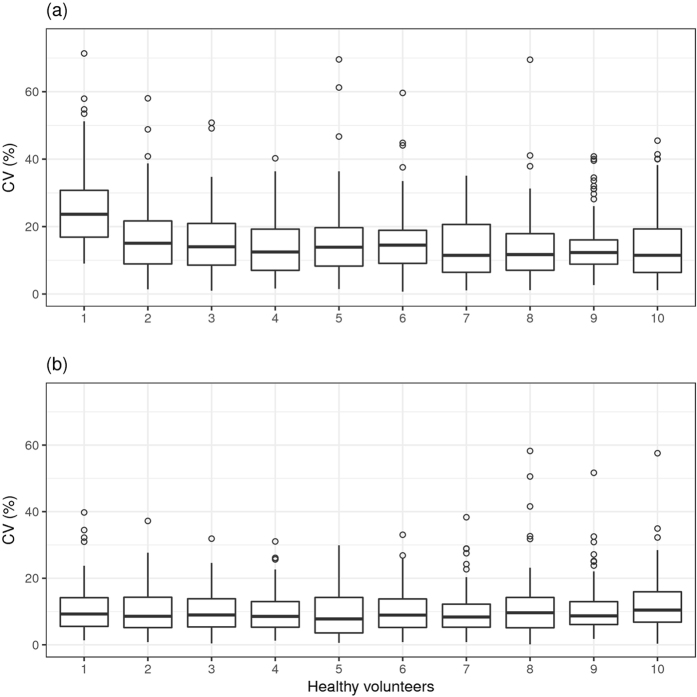
The ‘ten-sample’ set CVs for 81 peptide-transitions (56 proteins) after abundance ratio filter exclusions applied, common to both DBS and serum. (**a**) The CV for peptide-transition abundance ratios measured in DBS samples collected from ten healthy volunteers. (**b**) The CV for peptide-transition abundance ratios measured in serum samples from ten healthy volunteers. Note that the ten donors provided both DBS and serum samples and that the CV was based on three injections for each sample preparation.

The CVs for peptide-transition abundance ratios measured in DBS and serum samples from the single-sample set are summarized in Fig. 2 (Supplementary Table 2). The median CV across sample preparations was 6.50% in serum (range 5.90% to 6.84%; excluding the pooled sample) and 8.90% in DBS (range 8.19 to 9.93) samples. The CVs for peptide-transition abundance ratios measured in DBS and serum samples from the ten healthy volunteers (ten-sample set) are summarized in Fig. 3 and Supplementary Table 3. The median CV across the ten-sample set was 8.80% in serum (range 7.77% to 10.43%) and 13.18% in DBS (11.47% to 23.98%) samples. The median CV in the DBS healthy volunteer samples was inflated by increased variation in the first DBS sample. Exclusion, this sample resulted in a CV of 12.47% (range 11.47% to 15.05%).

### Correlation between DBS and serum peptide-transition relative intensities

**Figure 4:**
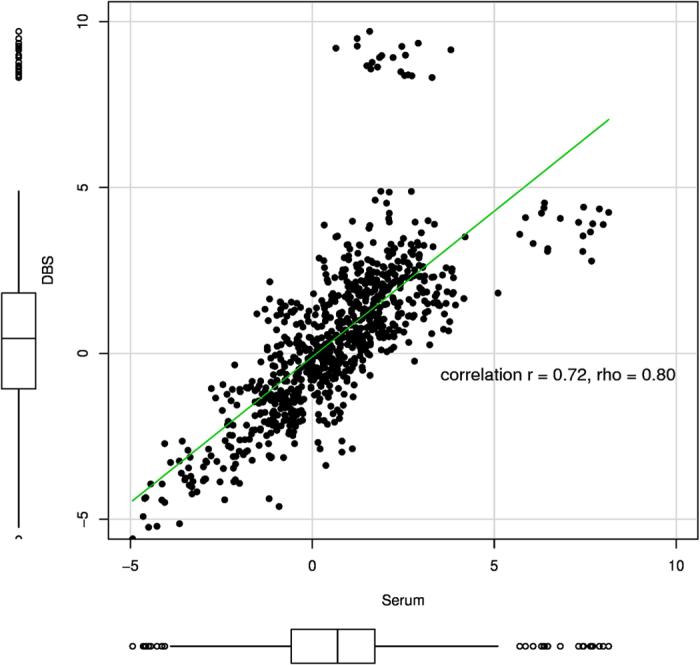
The ‘ten-sample’ set correlation between DBS and serum for 81 peptide-transitions (56 proteins) after abundance ratio filter exclusions applied, common to both DBS and serum. The correlation is based on the mean relative abundance ratios between DBS and serum samples from ten healthy volunteers. The DBS = b0 + b1serum regression line is plotted. The boxplots in the margins show the distribution of the DBS (y-axis) and serum (x-axis) abundance ratios. The figure was plotted using the R package car^41^.

The correlation coefficients between DBS and serum mean peptide-transition abundance ratios was strong (*r* = 0.72 and *rho* = 0.80 Fig. 4). This strong correlation indicates a relative consistencies between DBS and serum peptide-transition abundances. The correlation plot contains two clusters of peptide-transitions that stand out from the observed linear trend. The first cluster has higher relative abundance in DBS compared to serum and consists of one peptide transition from Apolipoprotein A-I (APOA1) and one from Ig gamma-1 chain C region (IGHG1). The second cluster has higher relative abundance in serum compared to DBS and consists of two peptide transitions from Albumin (ALBU).

## Discussion

MRM assays offer great sensitivity and specificity for protein quantification over a wide dynamic range of concentrations. The design of MRM experiments starts with the selection of unique prototypic peptides for each targeted protein. The selection of qualifier and quantifier transitions for each peptide is defined by several criteria including precursor charge states, fragment ion type, MS resolution and ionization. MRM based quantification methods require extensive method development to identify the most specific and sensitive transitions for each targeted peptide. As transitions are highly sensitive to the complexity of the biological matrix, interference free transitions have to be identified for each matrix. The increased specificity provided by SIS peptides offers a great advantage over semi-quantitative protein peptide assays. In addition, SIS peptides can also be implemented to normalize MS runs and minimize non-biological variation.

MRM method refinement requires the MS data to be visually checked for each targeted analyte in each given matrix to minimize interferences. Statistical approaches can complement the subjective and laborious process of transition filtering, improving the quality and reproducibility of the MS data. Unlike immunoassays in which quantification is at the protein level, MRM based proteomics studies monitor peptides by targeting specific transitions. As peptide-centric quantification strategies strongly rely on both protein digestion and peptide ionization efficiencies, selection of a representative feature becomes critical when breaking down proteins into peptides and further into transitions. From this perspective, reconstructing protein abundances from peptides and transitions can be challenging and requires further investigation.

Published research has approximated protein level analysis by analysing the following molecular surrogates: (1) summation of a defined number of peptide-transition abundances^32^; (2) peptide-transition abundances as repeat measurements^7^; and, (3) most abundant peptide-transitions containing a y fragment that is free of interferences^33^. As the measurement error is higher for the lower abundant transitions, the ‘noise’ introduced in the first approach will depend on the targeted peptides and their transition abundances. For the second approach, whether or not a peptide-transition correlation filter is used (*e.g*. between-run interference score^7^), the peptide fragmentation efficiency will be an issue and noise will be introduced to the analysis. Finally, although not necessarily a surrogate for protein abundance, analysing the most abundant peptide-transition, as adopted here, has a number of advantages. The most important being that this transition is often the most robustly measured transition for a given peptide and consequently, if selected as a predictor disease biomarker would provide a robust prediction of disease risk. The CVs (Figs 2 and 3) clearly demonstrate the robustness of the most abundant peptide-transition measurements. This was further demonstrated by the relative consistency of measurements as indicated by the strong correlation between peptide-transition abundances measured in DBS and serum samples collected from the healthy volunteers. We note that not all peptide-transitions can be measured in both matrices. The main difference in the DBS and serum proteomes is likely to result from the cellular components in DBS derived from red and white blood cells^8^,^34^. To ensure MS data quality, the selection of accurate and robust transitions, application of appropriate statistical methods combined with the use of several QC samples throughout the run are essential. The use of isotopically labelled internal standards is equally important for large scale studies as it facilitates method optimization, improved transition specificity and normalization to minimize non-biological systematic variation across the MS runs. Normalization is particularly important for proteomics based biomarker studies which can be conducted over a number of weeks. For instance, in a study involving 500 clinical samples and additional QC samples to verify the sample processing, with tens of proteins profiled over a 60 min gradient and three replicate injections are performed for each sample, the total run-time for such a study can take up to three months. It is very challenging to maintain the same instrument sensitivity through several months of run time. Therefore, implementing replicate QC sample injections through the run and using internal standards aid in monitoring instrument variations in large-scale clinical studies. However, in the absence of repeat sample injections, the total run-time can be reduced to 1 month. This example highlights the trade-off between the number of samples, the number of runs per sample (*i.e.* the accuracy of the measurement; technical replicates) and the study run-time. As proteomic based biomarker studies increase in size, robust and reproducible assays allowing single injections will be essential to achieve reasonable throughput without compromising data quality. As we have demonstrated good reproducibility of relative peptide abundance ratios measured with a median CV of 8.8% in serum and 13.2% in DBS samples, a single run per sample will be sufficient. Consequently, study run-time can be substantially reduced.

DBS quality is one of the most significant limitations for quantitative proteomics studies. For example, insufficient sample (small spot size) and multiple-spotting will both affect the protein content. We found, based on two DBS pilot studies, clear sample collection instructions improved DBS quality (data not shown). In this study, the reproducibility suggested that DBS discs have comparable protein abundances. However, despite recent studies showing that many proteins are stable in DBS^9^,^22^, the long term storage effects on protein stability and abundance in DBS have yet to be established. In this respect, the use of non-human internal standard proteins to normalize total protein concentration offers a potential solution to address this issue in future studies of large-scale clinical cohorts derived from multiple clinical centres with varying collection dates.

The combination of minimally invasive DBS sample collection combined with a highly specific and sensitive mass spectrometry technique allows targeted quantification of a large number of proteins in a single MS run. This approach has the potential to fundamentally change clinical proteomics and personalized medicine by facilitating large-scale studies.

## Methods

### Study design and sample preparation

All participants were healthy volunteers enrolled under protocols approved by the University of Cambridge Human Biology Research Ethics Committee. The study protocol was carried out in accordance with guidelines approved by the committee. All subjects gave informed written consent and all clinical investigations were conducted according to the principles of the Declarations of Helsinki. Two sample sets were used to select interference free quantifier transitions and to assess the reproducibility of the peptide-transition abundance measurements. The first set of samples (subsequently referred to as the ‘single-sample’ set) was used to evaluate the degree of variation of the peptide-transitions across different sample preparations of the same sample using the coefficient of variation (CV). It consisted of replicate preparations of two matrices: (1) ten DBS discs collected from the same healthy volunteer; and, (2) ten aliquots of reference Sigma serum (S7023, Sigma-Aldrich, Gillingham, U.K). DBS and sigma samples were run separately on the MS. Sample preparations were randomized to a well-plate position and eight consecutive injections were taken from each well. The second set of samples (subsequently referred to as the ‘ten-sample’ set) was used to evaluate the correlation between relative abundance measurements in DBS and serum. It consisted of DBS and serum samples collected at the same time from ten healthy volunteers. Samples were randomized in a well-plate position and three consecutive injections were taken from each well. The ten-sample set was also used to evaluate the degree of variation of the peptide-transitions within sample.

Sample collection, semi-automated protein digestion and peptide quantification methods are briefly summarized below and the details of the procedures are in the Supplementary Material. Protein extraction and digestion of DBS and serum samples were performed in a 96-well plate format using a liquid handling robotic system (Fig. 1). Trypsin-digested peptides were separated by C18 reverse phase chromatography prior to dynamic MRM-MS detection.

## MRM-Assay Development

### Targeted Protein-Peptide Selection

Eighty-two proteins were selected for targeted proteomics analysis. Unique peptides, surrogates of the targeted proteins, were filtered using Protein Basic Local Alignment Search Tool (BLAST) (http://blast.ncbi.nlm.nih.gov/Blast.cgi). Candidate peptides were then further filtered using the criteria listed below to reduce potential sources of variability. Peptides containing less than six amino acids (aa) were avoided to ensure uniqueness. Exclusion criteria also included large peptides ( > 20aa), potential ragged end peptides, peptides with known post-translational modifications (*e.g.* glycosylation and acetylation) and peptides containing missed tryptic cleavage sites (*e.g.* internal lysine or arginine residues) to prevent poor digestion and/or variable ionization efficiencies. Exceptions were made when no alternative peptides were available for a given candidate protein^6^,^33^. All reference SIS peptides with a C-terminal 13C- and 15N-labeled arginine (R) or lysine (K) were purchased from JPT (JPT Peptide Technologies, Berlin, Germany) for each endogenous peptide and used as an internal standard.

### Transition selection and interference screening

Ionization and fragmentation behaviours of all candidate peptides were extensively studied to select robust and interference-free peptide-transitions. A minimum of ten transitions, for each given peptide, for doubly and/or triply charged precursors in the range of 400–1200 Da were calculated using Skyline (version 3.1.0)^35^. All transitions were screened through the gradient to identify predominant charge state precursor-transition pairs. The preferred transitions were defined as follows: transition m/z greater than precursor m/z (to yield highest selectivity) and singly or doubly charged y ions. The b ions and transitions close to precursors were avoided to achieve the highest selectivity. Only peptides and transitions with maximum intensities and the highest spectral library similarity (dotp) were selected via Skyline using discovery proteomics data and available spectral data (Human NIST spectral library). Transition refinement and interference screening were performed on (i) SIS peptide mix in buffer, (ii) crude digested Sigma serum and pooled DBS samples, and (iii) Sigma serum and pooled DBS samples spiked with SIS peptide mix. The relative intensities of each of the targeted peptide-transitionswere compared for each matrix. The ion intensities of the endogenous (also known as ‘light’) peptides and the SIS (also known as ‘heavy’) peptides were adjusted to be within a 10-fold ratio for each peptide.

Three to four interference free transitions yielding the highest intensities and lowest noise were manually selected for DBS and serum, separately. Candidate transitions were then further evaluated using statistical approaches as described below.

### LC-MS/MS Analysis

An Agilent Infinity 1290HPLC and an Agilent 6495 QQQ LC/MS system with Agilent Jet Stream technology were utilized for peptide separation and detection. The separation was carried out on an Agilent AdvanceBio Peptide Map column (2.1 × 150 mm 2.7-micron) at 50◦C. Peptides were eluted over a linear gradient from 3% to 30% acetonitrile in 0.1% formic acid over 45 minutes. The MS was operated in positive mode. Instrument parameters including collision energies (CE) were then optimized to yield the highest sensitivity for all peptides and transitions.

Three to four interference-free transitions were selected for each target protein peptide as described above. Endogenous and corresponding SIS peptide-transitions were monitored and acquired simultaneously at unit resolution (0.7 Da) both in the first and third quadrupole (Q1 and Q3). Retention time (Rt) of each peptide was identified using full scan data. The final dynamic MRM method included 82 proteins, 156 peptides. Delta Rt window was 0.8 minutes and cycle time was 1 s. The minimum dwell time was 10 ms.

### Data pre-processing and quality control

Raw MS files were processed using the Skyline software package (Version 3.1.0). Peaks were manually checked, and peak integrations were adjusted accordingly where necessary. The endogenous peptide to internal standard SIS peptide peak area values were exported as a comma delimited data file for statistical analysis.

### Statistical analysis

Statistical analysis was conducted using the software package R (Version 3.2.3)^36^. As the problems of interference and ion suppression depend on the biological matrix^29^, we pre-processed and applied quality control measures to DBS and serum MS data separately. The order of the statistical pre-processing was as follow: (1) peptide-transition exclusions; (2) normalization; and, (3) log transformation of the peak area ratio.

### Normalization

We performed normalization based on the internal standard to minimize non-biological, systematic variation (technical variation) across MS runs. In other words, normalization was required to make peptide-transition abundances comparable across MS runs. Normalization can minimize differences in sample total protein amounts and technical variations in chromatography, for example. We adopted a median scaling normalization approach, in which individual transition abundances were multiplied by a ratio of the median internal standard abundance across all MS runs divided by the median internal standard abundance per MS run. This normalization can only correct for global MS shifts across runs and does not compensate for biological variation (interference and ion suppression) effects on individual peptide-transitions.

### Peptide-transition relative quantification

Relative peptide quantification was based on the relative abundance of the endogenous peptide-transitions compared with those of the respective internal standard, also known as the peak area (light/heavy isotope) ratio. The abundance ratio was used to minimize any remaining technical variation.

### Variance stabilization

As the variance of biological measurements often increases with intensity, we applied a log_2_ transformation, which is commonly used as a variance stabilising transformation as the variation of the logged abundances is less dependent on the absolute magnitude; skewed distributions become more symmetric and the influence of high-abundance transitions is reduced^37^.

### Identification of inaccurate peptide-transitions

As we wanted to reduce the amount of time spent on manually checking the peptide-transition peaks to identify inaccurate and imprecise peptide-transitions, we initially considered the following approaches: (i) the ‘between-run interference score’^7^; (ii) the all-pairs method^29^; (iii) peptide-transition rank comparison between the endogenous and the internal standard; and, (iv) peptide-transition abundance proportion comparison between the peptide and respective internal standard. Inaccurate peptide-transitions were flagged and visually checked to establish the utility of the tests.

The between run interference score (i), as defined by Surinova *et al*.^7^ and based on the endogenous peptide-transitions only, represents the correlation across the MS runs, between individual run peptide-transition abundance and the mean of their transition abundances for the corresponding peptide. We flagged peptide-transitions with a correlation coefficient *r* < 0.80 (ref. 7).

In the all-pairs approach (ii), as defined by Abbatiello *et al*.^29^, the abundance ratios were estimated for all possible transition pairs within a peptide and then compared between the endogenous peptide and respective internal standard. It was assumed that the abundance ratios should not differ between them. We tested at the peptide-transition-level whether the abundance ratios were significantly different between the peptide and respective internal standard. Rather than using a paired *t*-test and combining the *P*-values for each transition^29^, we used a random intercept linear mixed model to analyse all the pairs for a given peptide-transition, the random intercept allowing for differences between the healthy volunteer samples. We used the Benjamini-Hochberg false-discovery rate method to correct the *P*-values for multiple testing^38^.

In the rank approach (iii), for each individual MS run, we ranked the peptide-transition abundances within a peptide and required 80% rank consistency across the MS runs between the peptide and respective internal standard. Finally, in the proportion approach (iv), for each individual MS run, we simply calculated the peptide-transition proportion of the total peptide-transition abundance and tested for differences between the peptide and respective internal standard using a paired *t*-test, correcting the *P*-value for multiple testing as above.

### Coefficient of variation

We used the coefficient of variation (CV), which describes the amount of variability relative to the mean, to quantify the degree of variation for the peptide-transitions across the MS runs. For log_2_ transformed data, the geometric CV = 
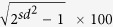
 (ref. 39), where sd is the standard deviation of the log-transformed data. Note that it is important to estimate the CV on the original untransformed scale of measurement.

### Correlation between DBS and serum peptide-transition relative abundances

Peptide-transition abundance in DBS and serum samples taken at the same time are unlikely to agree exactly due to the differences in the biological matrix. We used the ten-sample set to estimate correlation between DBS and serum. As we had three consecutive MS runs (injections) for each volunteer in the ten-sample set, we used the mean of the three relative peptide-transition abundances.

To measure the overall strength of the association between peptide-transition abundances derived from the two biological matrices, we calculated the Pearson product-moment correlation coefficient (*r*) and Spearman’s rank correlation coefficient (*rho*) between their relative abundances for the most abundant peptide-transitions. As the most abundant peptide-transition can vary with the biological matrix, we correlated the most abundant peptide-transitions common to both DBS and serum peptide-transition (95%) consistent between matrices. Perfect correlation (*r* = 1) between the abundance measurements does not necessarily indicate agreement between the measures, but rather that the points lie along any straight line. We only have perfect agreement when the points lie along the line of equality^40^.

## Additional Information

**How to cite this article**: Ozcan, S. *et al*. Towards reproducible MRM based biomarker discovery using dried blood spots. *Sci. Rep.* **7**, 45178; doi: 10.1038/srep45178 (2017).

**Publisher's note:** Springer Nature remains neutral with regard to jurisdictional claims in published maps and institutional affiliations.

## Supplementary Material

Supplementary Materials

## Figures and Tables

**Table 1:** A summary of the number of peptide-transitions flagged as ‘inaccurate’ based upon the four published approaches for the detection of inaccurate transitions.

		Inaccurate peptide-transitions (% of total)			
				Between run interference score	All-pairs	Rank	Proportion
Serum	Flagged	14 (3.8%)	259 (69.4%)	91 (24.4%)	233 (62.5%)
	Correctly flagged out of the 7 (1.9%) identified visually	0	7	7	7
DBS	Flagged	66 (17.7%)	218 (58.4%)	128 (34.3%)	192 (51.5%)
	Correctly flagged out of the 10 (2.7%) identified visually	1	5	8	7
Consistently flagged peptide-transitions between DBS and serum		8 (2.1%)	174 (46.6%)	85 (22.8%)	141 (37.8%)

The threshold correlation coefficient for the between run interference score was 0.8. Total number of peptide-transitions was 373.
