# Supplementary material for: Towards reproducible MRM based biomarker discovery using dried blood spots
Source: Sci Rep. 2017 Mar 27;7:45178. doi: 10.1038/srep45178 (PMC5366927; doi:10.1038/srep45178)
Supplement: Supplementary Materials [file srep45178-s1.pdf]

Supplementary Material for

"Towards reproducible MRM based biomarker discovery using dried blood spots"

Sureyya Ozcan, Jason D. Cooper, Santiago G. Lago, Diarmuid Kenny, Nitin Rustogi, Pawel Stocki, Sabine Bahn

Contents:

S1. Supplementary Methods.. P1

S1.1 Serum and DBS collection...P1

S1.2 Semi-Automated Protein Extraction and Digestion

Serum Digestion.. P2

DBS Digestion...P2

S2. Supplementary Tables.. P5

S1. Supplementary Methods

S1.1 Serum and DBS collection

Blood samples were collected from all subjects into BD Vacutainer Plus Plastic Serum Tubes (BD Vacutainer Systems, Plymouth, UK). Serum was then prepared by keeping samples at room temperature for 2 hours to allow blood coagulation, followed by centrifugation at 1,100 X g for 15 minutes. The resulting supernatants were stored at -80°C in Low Binding Eppendorf tubes (Hamburg, Germany) prior to analysis. DBS were collected from a finger prick, single blood drops were spotted onto Whatman 903 Protein Saver cards (Whatman, Dassel, Germany). A finger was first cleaned with 70% isopropyl alcohol and pricked with a sterile lancet. The first drop of blood was discarded using a cotton ball, and subsequent drops were collected onto the filter paper and air dried for at least 3h at room temperature. The DBS samples were stored at room temperature in a sealed storage bag containing a desiccant prior to analysis.

S1.2 Semi-Automated Protein Extraction and Digestion

A Biomek NX liquid handler (Beckman Coulter, High Wycombe, UK) was used to prepare both serum and DBS samples in a 96-well plate format.

Serum Digestion.

Aliquots of 5 µL of serum sample were dispensed into a Low-Bind Eppendorf 96-well plate containing 105 µL 50 mM ammonium bicarbonate. The protein extraction was followed by disulphide bond reduction [in the presence of 32.5 mM dithiothreitol (DTT) in 50 mM ammonium bicarbonate for 30 minutes at 60°C] and cysteine alkylation [with 75mM Iodoacetamide (IAA), in 50 mM ammonium bicarbonate for 30 minutes at room temperature in the dark]. Serum samples were then digested overnight (17 hours) using trypsin (Promega Corp, Madison, WI, USA) at a ratio of 1:50 (w/w) of enzyme to protein.

DBS Digestion.

The 3mm DBS discs were punched from the centre of the collection card using a manual puncher (Harris Micro-punch) and transferred into a Low-Bind Eppendorf 96-well plate. Proteins were extracted from the DBS samples using 40µL of 50 mM ammonium bicarbonate, dispensed by using a liquid handler, at 60°C for 30 minutes with gentle shaking. Protein reduction was performed using 18.3 mM DTT in 50 mM ammonium bicarbonate (at 60°C for 30 minutes), followed by alkylation with 46.6 mM IAA in 50 mM ammonium bicarbonate (30 minutes at room temperature in the dark). Sequencing grade modified trypsin was then added at a ratio of 1:20 (w:w) of enzyme to protein and incubated overnight (17 hours) at 37°C. Peptide enrichment and purification was performed using C-18 microfilter 96-well plates (FNSC18 from Glygen Corp., Columbia, MD, USA) on a Beckman Coulter BioMek NX workstation. For all the Solid Phase Extraction (SPE), cartridges were pre-conditioned and equilibrated with 100µL 0.1% formic acid (FA) (v/v), 100µL 0.1% acetonitrile (ACN) (v/v), and 100µL 0.1% FA (v/v), respectively. The trypsin digest (50µL) was loaded onto the cartridge and washed with 500µL 0.1%FA (v/v) (100µLx5 cycles). Elution was done with 60% acetonitrile (v/v). The eluent was then evaporated under vacuum and re-suspended in 50 µL of LC-MS mobile phase A (water containing 0.1% FA).

S2. Supplementary Tables

Supplementary Table 1. The number of peptide transitions by status based on the four approaches for the detection of inaccurate transitions for serum and DBS samples. Status: 0 – peptide transition excluded in all four approaches; 1 – passed by one approach; 2 – passed by 2 approaches; 3 – passed by 3 approaches; 4 – passed by all 4 approaches.

	DBS						
		0	1	2	3	4	Row total
Serum	0	3	5	4	0	0	12
	1	2	17	17	8	2	46
	2	0	8	41	17	28	97
	3	0	17	17	29	14	77
	4	0	0	31	16	134	181
	Column total	5	47	110	70	178	410

Supplementary Table 2(a) DBS peptide transition sample preparation variability (geometric CV by control sample preparation). SP – sample preparation.

Protein	Peptide	SP1	SP2	SP3	SP4	SP5	SP6	SP7	SP8	SP9	SP10	Median
A1AT	SVLGQLGITK	5.93	6.5	5.3	8.56	6.31	4.54	7.19	6.33	2.95	9.83	6.32
A1BG	ATWSGAVLAGR	5.31	8.9	8.68	10.7	9.05	14.16	11.76	10.43	12.75	13.83	10.56
A1BG	CLAPLEGAR	7.31	8.84	6.61	6.81	4.76	6.41	5.78	4.92	7.36	6.95	6.71
A1BG	SGLSTGWTQLSK	13.83	22.07	18.47	21.47	12.69	23.36	27.78	13.43	13.93	23.09	19.97
A2AP	DFLQSLK	9.19	9.96	6.32	8.81	9.49	5.14	6.04	7.07	7.13	5.93	7.1
A2MG	NEDSLVFVQTDK	9.4	10.44	11.35	9.66	6.3	7.45	16.3	5.38	8.36	7.63	8.88
AACT	ADLSGITGAR	2.84	8.34	4.47	2.98	7	5.85	7.01	7.71	4.34	4.48	5.17
AACT	EIGELYLPK	9.94	9.2	9.09	14.57	5.56	7.06	10.91	4.11	7.96	9.52	9.14
AACT	EQLSLLDR	10.78	6.08	10.8	8.87	10.66	7.16	8.81	8.07	6.93	7.18	8.44
ALBU	AAFTECCQAADK	14.03	15.76	20.42	20.55	10.71	16.64	8.63	18.35	8.48	23.59	16.2
ALBU	ETYGEMADCCAK	21.13	14.1	21.23	18.01	19.19	20.06	10.59	19.84	9.93	14.36	18.6
AMBP	TVAACNLPIVR	13.07	9.36	12.27	8.54	11.01	11.29	6.44	7.21	12.47	12.25	11.15
ANGT	ALQDQLVLVAAK	2.57	7.23	9.57	10.27	8.4	5.76	9.55	8.25	7.83	8.94	8.32
ANGT	SLDFTELDVAAEK	12.53	6.52	8.59	9.99	9.04	8.91	9.05	8.15	6.01	8.33	8.75
ANT3	LPGIVAEGR	15.33	14.75	13.64	14.12	9	11.17	9.82	11.49	6.71	11.51	11.5
APOA1	ATEHLSTLSEK	20.59	16.72	14.69	12.65	15.44	19.16	13.14	17.26	11.96	12.02	15.06
APOA2	SPELQAEAK	3.89	3.46	3.51	2.59	3.59	4.59	3.67	4.15	1.97	4.08	3.63
APOA4	ALVQQMEQLR	11.91	13.02	18.4	11.62	11.16	17.06	13.76	14.44	10.37	14.01	13.39
APOA4	IDQNVEELK	12.84	10.29	14.05	15.7	9.45	10.17	5.17	7.27	11.32	7.3	10.23
APOA4	ISASAEELR	4.89	3.13	1.97	4.32	7.29	4.62	5.46	3.25	4.03	4.03	4.18
APOC1	EFGNTLEDK	4.64	6.82	6.58	8.61	5.45	8.53	7.82	4.99	7.1	7.61	6.96
APOC1	EWFSETFQK	17.53	12.88	18.29	14.7	17.65	15.33	27.72	14.9	15.94	16.7	16.32
APOC2	TAAQNLYEK	10.14	9.88	4.89	7.97	10.49	10.73	6.97	9.33	7.99	10.97	9.61
APOC3	GWVTDGFSSLK	10.74	4.06	5.17	6.79	6.6	7.09	6.45	3.94	4.68	3.57	5.81
APOC4	AWFLESK	12.84	10.8	13.29	10.08	8.3	6.69	14.8	15.89	13.84	7.96	11.82

APOD	VLNQELR	7.6	6.1	4.34	2.64	2.75	5.87	5.9	3.36	1.88	6.18	5.11
APOE	AATVGSLAGQPLQER	13.79	4.86	10.09	11.63	9.63	7.99	12.37	7.15	8.1	6.63	8.87
APOE	LGPLVEQGR	5.01	11.2	5.95	9.36	8.62	8.05	9.01	9.14	6.47	5.95	8.34
APOE	SELEEQLTPVAEETR	8.21	10.94	21.9	12.93	12.55	12.38	16.14	8.57	10.53	12.58	12.46
APOH	VSFFCK	11.17	9.65	11.62	13.47	13.83	9.1	6.55	12.87	7.67	8.95	10.41
APOL1	VTEPISAESGEQVER	27.05	20.77	14.88	16.54	20.18	15.83	25.05	25.96	27.17	17.05	20.48
APOM	AFLLTPR	7.35	4.76	9.82	5.02	8.66	6.46	13.68	6.65	4.57	5.6	6.55
APOM	SLTSCLDSK	7.65	3.69	9.91	8.93	5.6	9.08	15.64	9.52	15.05	8.01	9
C1R	YTTEIIK	18.17	11.36	16.3	15.18	7.57	10.8	6.09	9.08	15.94	9.24	11.08
C1S	LLEVPEGR	5.33	4.93	5.53	6.89	5.65	2.32	6.35	5.22	6.47	8.69	5.59
C1S	TNFDNDIALVR	29.04	10.96	28.39	14.47	17.86	38.38	11.44	12.95	10.43	26.73	16.16
C4BPA	EDVYVVGTVLR	9.88	7.3	4.25	6.4	6.54	6.23	8.11	6.99	11.97	6.87	6.93
C4BPA	YTCLPGYVR	22.09	22	23.78	21.74	18.4	19.97	20.71	16.35	27.03	15.21	21.23
CBG	GTWTQPFDLASTR	9.91	5.05	10.22	9.25	8.74	7.7	11.94	6.23	8.73	9.66	9
CBPB2	DTGTYGFLLPER	7.42	13.9	14.8	6.03	9.18	11.33	7.14	5.47	12.79	8.09	8.63
CERU	EVGPTNADPVCLAK	13.4	11.19	9.62	9.56	12.26	13.64	14.17	7.5	7.41	13.11	11.72
CFAB	DISEVVTPR	7.88	9.21	8.57	7.86	5.38	11.67	5.63	7.13	9.7	7.34	7.87
CFAB	DLLYIGK	8.67	4.59	5.27	3.62	3.97	5.02	8.61	6.71	6.85	4.42	5.14
CFAB	EELLPAQDIK	7.33	9.44	5.67	9.21	7.39	7.23	5.29	8.22	4.19	6.28	7.28
CFAB	YGLVTYATYPK	21.41	15.42	15.17	10.78	14.5	14.22	14.25	9.51	12.2	13.8	14.23
CLUS	IDSLLENDR	8.29	9.1	5.39	8.77	8.04	6.62	7.94	7.93	4.33	6.21	7.94
CO4A	DFALLSLQVPLK	12.9	18.06	15.18	12.85	15.21	18.15	24.11	11.44	15.28	15.62	15.25
CO4A	ITQVLHFTK	13.73	4.21	7.81	5.67	9.73	7.4	5.89	10.47	5.89	7.8	7.6
CO8A	MESLGITSR	11.93	8.47	7.3	4.78	5.3	6.33	9.8	7.45	5.67	7.29	7.29
CO9	LSPIYNLVPVK	17.23	19.22	21.71	9.11	13.45	15.73	24.18	30.7	25.35	22.19	20.46
CO9	VVEESELAR	17.3	7.02	12.41	8.3	8.84	10.94	11.03	11.52	7.21	11.11	10.98
FA12	VVGGLVALR	5.41	10.92	9.07	7.67	7.48	4.62	7.6	3.84	5.8	6.56	7.02
FETUA	FSVVYAK	3.81	6.65	7.49	3.88	6.1	7.58	6.65	3.13	4.81	3.7	5.46
FETUA	HTLNQIDEVK	7.8	6.38	5.74	6.8	5.54	10.37	5.05	8.55	6.37	4.88	6.38
FINC	YSFCTDHTVLVQTR	20.2	16.95	14.15	24.03	17.73	21.88	23.64	11.14	32.71	17.13	18.96
GELS	AGALNSNDAFVLK	12.63	13.05	14.15	7.99	8.4	17.38	7.64	5.46	13.27	9.69	11.16

GELS	SEDCFILDHGK	29.84	38.48	32.04	22.1	18.19	29.34	22.11	41.02	23.36	18.99	26.35
HEMO	NFPSPVDAAFR	2.59	2.27	3.14	1.41	4.67	3.79	4.03	2.66	3.07	3.38	3.1
HEMO	VDGALCMEK	4.14	10.05	8.09	4.9	6.07	9.1	6.29	7.68	5.72	3.82	6.18
HEP2	FAFNLYR	6.57	8.97	8.74	7.01	8.06	9.01	4.97	7.73	7.57	8.49	7.9
HEP2	IAIDLFK	4.2	3.48	4.03	4.99	4.19	4.48	2.75	3.34	4.19	4.69	4.19
HPT	VGYVSGWGR	4.22	4.84	6.88	4.79	4.81	5.58	5.52	11.23	4.23	5.22	5.03
HPT	VTSIQDWVQK	10.74	6.51	6.7	12.87	9.73	5.27	9.39	7.65	10.33	13.24	9.56
IC1	FQPTLLTLPR	2.75	4.59	2.52	3.18	3.1	2.18	4.04	2.79	2.97	2.78	2.88
IGHA1	TPLTATLSK	4.41	2.89	5.28	3.72	3.42	2.27	3.76	2.86	3.08	4.7	3.57
IGHG1	FNWYVDGVEVHNAK	15.13	7.79	12.01	9.07	9.34	6.73	13.11	10.89	7.19	15.4	10.12
IGHG3	NQVSLTCLVK	6.32	4.14	4.82	4.13	5	6.66	3.7	5.9	4.72	3.66	4.77
IGHM	QIQVSWLR	4.33	4.59	3.05	4.13	4.04	2.98	2.49	2.24	2.49	3.85	3.45
IGHM	YAATSQVLLPSK	6.76	6.95	11.11	8.38	9.74	6.15	5.75	9.54	3.83	8.68	7.67
ITIH1	LDAQASFLPK	11.47	16.29	11.5	10.51	10.6	14.63	7.46	11.01	12.98	9.29	11.24
ITIH2	FYNQVSTPLLR	11.38	10.21	14.36	16.14	10.7	11.88	7.11	7.53	6.8	11.28	10.99
ITIH2	IQPSGGTNINEALLR	8.59	6.5	4.56	12.91	7.89	4.47	8.54	8.8	5.98	7.3	7.59
ITIH4	ETLFSVMPGLK	12.49	9.67	15.04	7.82	13.99	9.72	7.55	12.17	10.33	7.06	10.03
KNG1	DFVQPPTK	2.74	3.94	5.34	4.92	6.54	3.68	4.57	5.05	5.39	4.17	4.75
LUM	SLEDLQLTHNK	14.64	9.6	10.64	18.17	8.84	9.24	12.35	10.83	14.84	12.61	11.59
PEDF	TVQAVLTVPK	6.6	13.66	16.34	7.63	9.43	7.37	10.35	14.64	7.51	12.29	9.89
PGRP2	TFTLLDPK	10.4	4.22	12.32	8.42	12.78	10.13	8.76	12.15	10.48	9.88	10.27
PHLD	NQVVIAAGR	16.35	6.85	10.68	9.19	9.5	8.27	10.04	13.84	14.46	9.13	9.77
PLMN	FVTWIEGVMR	7.47	1.95	4.25	6.2	7.55	4.81	6.87	4.74	4.76	5.47	5.14
SAMP	IVLGQEQDSYGGK	18.87	6.6	11.39	19.4	11.96	11.96	13.12	20.57	7.2	11.18	11.96
THRB	ELLESYIDGR	10.13	10.85	9.13	9.95	11.59	16.61	11.75	9.57	7.09	10.59	10.36
TRFE	EGYYGYTGAFR	12.2	14.01	7.65	12.71	12.37	10.5	14.77	6.64	8.96	8.18	11.35
TTHY	AADDTWEPFASGK	22.67	9.19	14.84	22.74	21.69	18.51	14.84	20.02	12.72	14.26	16.68
TTHY	VLDAVR	5.58	5.02	7.87	7.46	4.71	10.2	6.97	7.61	5.6	7.9	7.21
VTNC	DVWGIEGPIDAAFTR	7.66	10.89	16.3	9.2	11.48	13.27	11.1	10.09	8.88	9.63	10.49
VTNC	DWHGVPGQVDAAMAGR	8.25	9.41	5.28	11.41	6.37	10.84	8.81	9.93	4.92	6.95	8.53

Supplementary Table 2(b) Serum peptide transition sample preparation variability (geometric CV by control sample preparation). SP – sample preparation.

Protein	Peptide	SP1	SP2	SP3	SP4	SP5	SP6	SP7	SP8	SP9	SP10	Median
A1AT	SVLGQLGITK	9.3	5.47	4.67	6.45	6.38	6.04	4.3	6.71	5.81	1.96	5.92
A1BG	ATWSGAVLAGR	12.04	10.3	6.64	6.63	5.04	9.4	12.95	7.66	1.84	13.58	8.53
A1BG	CLAPLEGAR	3.94	5.79	5.29	5.94	4.96	4.82	6.68	4.02	4.38	3.82	4.89
A1BG	SGLSTGWTQLSK	10.18	10.36	9.72	15.15	11.39	13.09	17.94	10.37	14.94	10.24	10.88
A2AP	DFLQSLK	6.79	8.61	5.63	7.01	8.39	8.03	7.37	6.62	5.15	5.37	6.9
A2MG	NEDSLVFVQTDK	8.94	9.13	7.96	6.12	4.96	5.93	6.94	6.41	6.54	7.9	6.74
AACT	ADLSGITGAR	4.48	4.25	7.69	3.03	7.75	3.71	4.58	6.66	8.75	9.12	5.62
AACT	EIGELYLPK	8.35	7.32	8.68	5.64	5.43	6.09	7.78	6.1	5.03	6.73	6.42
AACT	EQLSLLDR	6.35	6.34	5.07	5.52	6.68	6.95	7.74	5.55	10.27	11.22	6.51
ALBU	AAFTECCQAADK	9.61	9.73	7.48	10.55	9.45	2.56	6.8	8.07	12.74	6.49	8.76
ALBU	ETYGEMADCCAK	6.98	6.75	6.48	6.43	6.44	5.6	9.52	3.82	3.73	10.1	6.46
AMBP	TVAACNLPIVR	7.19	8.3	4.57	5.55	7.47	5.31	6.5	4.65	7.83	7.22	6.85
ANGT	ALQDQLVLVAAK	2.94	6.46	4.36	5.59	4.5	4.7	4.94	6.02	3.93	4.9	4.8
ANGT	SLDFTELDVAAEK	9.77	6.3	9.47	4.66	9.98	9.21	8.68	5.55	6.54	7.98	8.33
ANT3	LPGIVAEGR	11.76	8.39	4.65	9.93	10.53	8.42	13.42	8.64	9.14	6.78	8.89
APOA1	ATEHLSTLSEK	6.83	7.4	8.64	5.06	6.8	4.92	9.31	4.03	6.37	5.44	6.58
APOA2	SPELQAEAK	4.03	6.78	6.94	3.53	4.8	7.14	6.94	4.37	4.66	4.28	4.73
APOA4	ALVQQMEQLR	16.71	14.04	11.7	15.4	8.84	7.79	14.96	9.46	18.92	9.04	12.87
APOA4	IDQNVEELK	14.68	9.84	8.21	9.83	11.38	11.48	8.55	6.57	6.96	12.15	9.84
APOA4	ISASAEELR	3.01	3.68	4.79	3.44	4.31	2.61	4.12	2.6	3.18	5.05	3.56
APOC1	EFGNTLEDK	6.99	9.27	11.46	10.64	12.35	6.47	11.93	4.1	10.49	9.81	10.15
APOC1	EWFSETFQK	7.21	13.54	3.21	16.9	6.27	6.18	11.08	9.58	8.23	13.22	8.91
APOC2	TAAQNLYEK	4.15	9.73	7.62	6.32	7.68	4.91	4.76	7.49	7.47	6.38	6.92
APOC3	GWVTDGFSSLK	2.32	4.95	4	3.19	2.68	4.43	4.05	3.38	4.82	4.12	4.03
APOC4	AWFLESK	10.15	10.47	8.45	10.58	7.38	11.56	13.48	11.22	9.1	6.15	10.31

APOD	VLNQELR	3.07	6.11	4.26	2.54	4.52	2.9	4.8	2.71	2.8	4.39	3.66
APOE	AATVGSLAGQPLQER	8.26	8.62	6.39	7.8	6.2	5.53	6.62	14.18	8.55	10.07	8.03
APOE	LGPLVEQGR	3.11	3.82	3.21	3.5	3.2	3.32	3.05	3.7	2.08	3.03	3.21
APOE	SELEEQLTPVAEETR	8.9	5.57	7.82	7.14	7.69	7.46	9.18	10.27	11.3	8.06	7.94
APOH	VSFFCK	3.61	3.67	4.06	4.53	4.32	3.91	5.28	3.22	5.15	4.61	4.19
APOL1	VTEPISAESGEQVER	9.22	22.17	18.57	18.53	29.53	15.77	22.5	20.08	5.83	17.45	18.55
APOM	AFLLTPR	6.77	3.31	4.75	5.31	3.87	4.43	7.22	4.49	4.72	4.01	4.61
APOM	SLTSCLDSK	4.7	5.82	7.14	3.77	7.23	6.36	5.9	4.27	6.32	3.49	5.86
C1R	YTTEIIK	12	17.05	11.84	9.98	11.31	10.8	11.02	18.47	13.95	14.54	11.92
C1S	LLEVPEGR	1.85	4.95	6.88	5.25	7.45	6.24	5.16	5.95	9.27	7.61	6.1
C1S	TNFDNDIALVR	11.51	23.74	20.32	15.18	13.09	12.7	12.32	19.82	14.34	14.81	14.57
C4BPA	EDVYVVGTVLR	2.58	5.75	6.94	5.47	4.07	6.98	4.57	3.21	5.49	2.58	5.02
C4BPA	YTCLPGYVR	10.49	9.62	12.49	9.25	8.99	11.06	11.2	11.38	7.66	8.35	10.05
CBG	GTWTQPFDLASTR	8.45	5.67	8.35	10.74	6.74	8.54	8.83	5.1	9.77	5.26	8.4
CBPB2	DTGTYGFLLPER	15.24	11	16.91	11.03	14.02	12.19	16.25	14.86	12.29	9.73	13.15
CERU	EVGPTNADPVCLAK	12.4	9.75	10.02	7.48	11.13	7.62	9.19	6.97	9.72	14.14	9.73
CFAB	DISEVVTPR	5.46	5.4	5.08	5.11	5.29	3.62	7.32	4.85	5.95	6.38	5.35
CFAB	DLLYIGK	4.57	6.29	2.96	7.24	3.89	4.89	5.53	4.21	5.4	5.41	5.14
CFAB	EELLPAQDIK	7.07	9.53	5.06	6.78	4.87	6.26	7.59	3.87	9.34	6.76	6.77
CFAB	YGLVTYATYPK	16.53	13.33	5.87	10.55	12.16	15.55	12.96	11.34	13.82	9.97	12.56
CLUS	IDSLLENDR	5.87	7.87	9.09	11.38	12.53	8.1	7.23	7.6	6.12	6.4	7.73
CO4A	DFALLSLQVPLK	6.22	5.64	4.57	5.14	6.13	4.72	4.75	6.84	7.27	6.8	5.88
CO4A	ITQVLHFTK	4.41	5.46	8.49	8.48	5.17	5.52	8.41	4.73	7.83	6.71	6.12
CO8A	MESLGITSR	5.47	5.79	7.09	8	6.52	7.59	9.46	7.01	9.05	9.08	7.34
CO9	LSPIYNLVPVK	5.66	4.89	6.3	7.08	4.22	6.43	6.89	2.73	5.57	7.86	5.98
CO9	VVEESELAR	6.18	8.98	5.16	3.26	7.98	10.79	8.04	5.55	5.82	4.57	6
FA12	VVGGLVALR	8.28	7.27	5.34	4.83	5.5	3.22	4.84	10.87	2.04	7.24	5.42
FETUA	FSVVYAK	2.73	4.86	7.15	4.78	3.19	5.16	5.07	5.21	2.99	4.13	4.82
FETUA	HTLNQIDEVK	6.3	7.23	4.72	6.52	7.89	3.56	8.76	6.42	11.54	12.79	6.88
FINC	YSFCTDHTVLVQTR	11.19	21.25	11.69	15.88	12	12.28	13.72	14.52	16.01	15.67	14.12
GELS	AGALNSNDAFVLK	10	6.42	10.05	11.58	12.08	12.39	5.14	10.51	13.65	9.66	10.28

GELS	SEDCFILDHGK	20.56	17.33	16.44	6.66	13.34	15.56	16.22	12.19	7.64	10.38	14.45
HEMO	NFPSPVDAAFR	2.55	1.6	1.44	1.76	1.89	2.59	2.64	2.7	2.77	1.74	2.22
HEMO	VDGALCMEK	4.29	4.01	3.14	5.39	5.67	3.27	2.88	4.48	5	2.59	4.15
HEP2	FAFNLYR	1.67	2.83	1.73	1.64	2.16	1.93	1.58	2.3	2.03	2.4	1.98
HEP2	IAIDLFK	6.77	12.55	5.43	4.58	6.66	4.66	5.7	7.38	6.37	5.05	6.04
HPT	VGYVSGWGR	1.78	3.41	3.4	2.57	2.24	2.45	3.91	2.78	1.23	3.71	2.67
HPT	VTSIQDWVQK	7.86	8.26	4.27	5.48	4.63	5.73	4.8	8.56	3.53	7.06	5.61
IC1	FQPTLLTLPR	4.03	4.1	6.22	3.06	3.7	5.36	4.34	4.66	4.85	2.22	4.22
IGHA1	TPLTATLSK	1.34	1.32	3.1	1.89	1.98	3.2	3.13	3.41	1.19	2.3	2.14
IGHG1	FNWYVDGVEVHNAK	4.52	3.08	5.13	4.03	6.44	3.19	2.9	4.33	2.8	2.46	3.61
IGHG3	NQVSLTCLVK	4.12	6.49	4.53	4.54	1.76	7.56	4.33	4.51	4.15	3.89	4.42
IGHM	QIQVSWLR	3.73	4.97	6.17	4.44	4.9	5.8	7.27	6.29	3.94	4.51	4.94
IGHM	YAATSQVLLPSK	4.34	6.42	3.83	9.39	5.7	6.75	4.81	5.52	5.75	5.63	5.66
ITIH1	LDAQASFLPK	9.32	9.18	3.9	9.95	4.6	9.23	3.38	8.75	10.91	11.13	9.21
ITIH2	FYNQVSTPLLR	6.71	5.48	6.47	5.39	6.64	5.08	7.35	6.46	6.22	5.93	6.34
ITIH2	IQPSGGTNINEALLR	8.05	8.55	8.19	10.18	9.76	9.87	4.22	7.32	8.5	7.85	8.34
ITIH4	ETLFSVMPGLK	2.18	2.45	4.18	3.62	3.72	3.3	2.53	4.18	5.23	5.09	3.67
KNG1	DFVQPPTK	4.71	5.44	4.48	4.46	5.74	5.17	5.04	4.27	5.64	5.45	5.11
LUM	SLEDLQLTHNK	11.74	8.16	15.08	8.34	10.52	4.6	6	11.05	6.25	5.53	8.25
PEDF	TVQAVLTVPK	7.32	7.58	8.71	11.5	4.07	4.83	7.44	8.68	6.21	8.81	7.51
PGRP2	TFTLLDPK	4.64	6.04	4.78	6.16	10.96	4.99	4.84	8.22	5.41	3.78	5.2
PHLD	NQVVIAAGR	10.12	10.55	9.53	9.72	11.99	14.53	12.41	14.32	15.23	8.49	11.27
PLMN	FVTWIEGVMR	3.96	5.03	4.09	3.71	5.02	3.28	4.66	4.25	5.47	5.29	4.46
SAMP	IVLGQEQDSYGGK	25.85	16.11	9.4	9.61	19.15	15.53	6.65	21.88	20.66	16.35	16.23
THRB	ELLESYIDGR	5.23	8.34	7.71	4.04	5.71	4.07	3.59	4.83	4.81	7.2	5.03
TRFE	EGYYGYTGAFR	8.19	8.52	8.64	10.81	7.74	5.86	11.21	9.43	7.9	5.19	8.36
TTHY	AADDTWEPFASGK	23.95	26.4	15.53	24.68	15.93	19.36	10.95	16.51	18.7	21.01	19.03
TTHY	VLDAVR	5.42	2.52	6.44	4.3	3.85	6.71	6.3	4.5	4.35	2.5	4.42
VTNC	DVWGIEGPIDAAFTR	4.51	3.93	4.56	7.97	5.28	4.69	5.58	3.72	4.34	3.56	4.54
VTNC	DWHGVPGQVDAAMAGR	9.96	6.7	7.86	8.05	7	3.67	8.12	7.92	4.08	6.78	7.43

Supplementary Table 3(a) DBS peptide transition sample to sample variability (geometric CV by healthy volunteer sample). S – healthy volunteer sample.

**Protein**	**Peptide**	**S1**	**S2**	**S3**	**S4**	**S5**	**S6**	**S7**	**S8**	**S9**	**S10**	**Median**
A1AT	SVLGQLGITK	48.67	10.96	10.12	1.63	1.87	20.16	9.38	7.67	11.42	3.79	9.75
A1BG	ATWSGAVLAGR	16.99	5.99	3.5	4.37	5.25	17.7	3.43	17.89	16.05	9.83	7.91
A1BG	CLAPLEGAR	10.43	23.26	8.05	18.42	17.32	9.43	24.93	11.39	16.34	11.13	13.87
A1BG	SGLSTGWTQLSK	18.81	38.2	9.35	14.4	69.58	32.11	23.66	28.52	4.59	12.6	21.23
A2AP	DFLQSLK	28.58	10.7	12.14	3.3	1.56	0.71	11.93	16.11	7.89	8.22	9.46
A2MG	NEDSLVFVQTDK	14.55	13.29	10.11	19.25	8.3	14.44	21.83	19.73	12.78	9.54	13.86
AACT	ADLSGITGAR	22.51	2.26	12.15	5.34	10.46	14.82	15.89	9.57	9.58	7.65	10.02
AACT	EIGELYLPK	24.58	18.18	20.91	5.14	14.52	14.19	6.76	5.42	10.44	17.63	14.36
ALBU	AAFTECCQAADK	25.84	24.89	15.37	12.47	9.01	17.59	3.28	6.79	2.66	2.55	10.74
ALBU	ETYGEMADCCAK	21.35	8.91	16.44	13.62	3.91	31.06	28.94	12.43	13.63	45.48	15.04
AMBP	TVAACNLPIVR	22.01	6.04	23.61	5.69	22.28	16.04	21.61	8.66	12.57	25.25	18.82
ANGT	ALQDQLVLVAAK	51.21	29.9	1	30.54	6.19	17.32	8.24	21.65	8.95	8.22	13.13
ANGT	SLDFTELDVAAEK	48.95	14.01	10.79	15.74	8.45	15.08	5.18	17.68	8.43	10.7	12.4
ANT3	LPGIVAEGR	15.91	15.08	26.76	14.8	28.01	29.08	20.74	7.28	19.65	18.15	18.9
APOA1	ATEHLSTLSEK	22.72	24.61	8.16	21.74	23.59	3.17	17.94	21.97	13.67	11.48	19.84
APOA2	SPELQAEAK	28.63	12.83	15	9.58	7.8	9.09	11.47	13.41	7.45	22.7	12.15
APOA4	ALVQQMEQLR	42.03	34.92	50.82	8.91	61.26	22.74	14.96	24.71	39.56	40.09	37.24
APOA4	ISASAEELR	22.49	18.26	3.94	18.22	13.83	2.56	11.71	2	5.43	6.17	8.94
APOC1	EFGNTLEDK	24.54	15.6	6.72	21.68	19.37	5.81	14.88	11.01	10.13	4.12	12.95
APOC1	EWFSETFQK	29.6	31.56	14.29	29.89	9.91	10.99	14.12	15.76	40.04	6.71	15.02
APOC2	TAAQNLYEK	24.79	5.36	4.49	30.25	19.68	29.6	4.25	9.29	25.77	16.28	17.98
APOC3	GWVTDGFSSLK	36.98	13.7	18.61	10.19	14.15	8.03	23.9	6.82	4.59	5.06	11.95
APOC4	AWFLESK	10.38	17.4	22.57	40.25	20.44	14.9	11.41	11.69	2.92	11.53	13.29
APOD	VLNQELR	18.78	19.77	12.69	5.4	15.09	4.72	4.15	9.48	5.89	8.68	9.08

APOE	AATVGSLAGQPLQER	30.17	17.04	16.65	15.66	46.7	11.48	3.71	16.05	12.07	6.82	15.86
APOE	SELEEQLTPVAEETR	53.48	17.27	14.26	8.99	36.4	18.9	18.97	27.2	31.12	38.22	23.09
APOH	VSFFCK	16.49	17.79	21.43	8.12	16.22	7.06	23.87	3.27	28.15	24.85	17.14
APOL1	VTEPISAESGEQVER	30.62	40.82	29.56	4.37	14.73	44.08	24.68	12.94	17.39	38.25	27.12
APOM	AFLLTPR	13.51	8.23	3.26	10.35	7.58	2.97	7.18	3.51	13.56	6	7.38
APOM	SLTSCLDSK	27.82	48.84	26.64	13.47	11.63	19.28	12.58	17.95	6.37	23.34	18.62
C1R	YTTEIIK	30.22	6.35	25.08	13.12	13.89	16.2	10.05	11.61	12.76	7.41	12.94
C1S	LLEVPEGR	13.65	9.44	15.85	17.41	14.77	16.14	1.6	3.98	15.5	13.08	14.21
C1S	TNFDNDIALVR	33.14	10.33	12.44	23.9	8.85	9.92	20.63	8.83	13.62	14.61	13.03
C4BPA	EDVYVVGTVLR	39.55	16.53	5.05	8.83	10.2	9.59	8.19	14.91	9.63	11.49	9.91
C4BPA	YTCLPGYVR	26.94	15.05	20.13	12.44	9.76	18.2	10.4	3.09	24.39	13.22	14.14
CBG	GTWTQPFDLASTR	50.88	12.01	16.36	4.98	21.58	13.33	8.92	13.27	33.62	13.37	13.35
CERU	EVGPTNADPVCLAK	21.34	21.67	23.82	6.16	14.8	37.57	24.59	14.37	22.33	4.87	21.51
CFAB	DISEVVTPR	20.03	19.8	13.1	31.38	7.57	26.5	9.88	14.07	26.04	3.26	16.94
CFAB	DLLYIGK	21.9	1.41	6.44	2.2	6.12	2.4	14.26	2.29	9.29	4.51	5.31
CFAB	EELLPAQDIK	9.89	6.43	20.51	23.1	15.59	8.84	4.4	9.99	12.72	2.28	9.94
CFAB	YGLVTYATYPK	38.69	36.93	27.01	29.52	14.75	17.99	6.09	37.91	31.18	9.65	28.27
CLUS	IDSLLENDR	23.29	24.57	15.35	8.2	21.39	2.07	6.5	19.94	34.53	12.19	17.64
CO4A	DFALLSLQVPLK	42.6	14.15	21.81	21.18	17.84	7.16	3.87	17.9	5.74	22.33	17.87
CO4A	ITQVLHFTK	14.33	12.84	15.33	15.01	16.79	17.21	12.2	9.76	14.46	1.72	14.39
CO8A	MESLGITSR	28.91	3.21	34.73	14.79	4.54	20.8	1.1	12.3	12.14	26.02	13.54
CO9	LSPIYNLVPVK	54.77	8.06	13.54	8.85	19.23	9.7	6.43	3.66	5.3	15.12	9.27
CO9	VVEESELAR	14.76	7.63	2.37	36.4	19.1	3.38	6.47	28.99	11.27	7.97	9.62
FA12	VVGGLVALR	12.65	5.39	14.01	8.5	7.74	18.71	8.06	4.24	17.32	17.97	10.57
FETUA	FSVVYAK	12.27	10.95	2.24	16.39	9.11	6.74	7.04	2.91	13.92	16.61	10.03
FETUA	HTLNQIDEVK	12.16	8.77	2.88	7.19	6.07	14.97	5.06	8.11	8.8	5.54	7.65
GELS	AGALNSNDAFVLK	9.2	4.66	13.35	20.69	20.94	28.72	20.89	14.05	11.87	41.44	17.37
GELS	SEDCFILDHGK	23.98	10.6	12.1	20.31	5.7	17.56	7.63	13.38	14.2	11.65	12.74
HEMO	NFPSPVDAAFR	36.14	7.68	6	3.97	3.53	10.17	6.97	4.96	3.78	1.62	5.48
HEMO	VDGALCMEK	29.46	29.61	23.25	6.39	15.4	17.15	16.99	21.56	32.04	27.88	22.41
HEP2	FAFNLYR	25.22	11.16	15.09	3.46	13.85	15.04	2.44	16.12	12.29	8.19	13.07

HEP2	IAIDLFK	18	9.55	9.31	2.94	7.47	3.59	18.12	13.52	9.58	15.31	9.57
HPT	VGYVSGWGR	22.41	10.64	11.12	6.48	11.9	12.93	15.47	5.83	12.66	6.07	11.51
HPT	VTSIQDWVQK	25.05	19.05	4.08	15.87	7.74	1.06	21.13	21.76	13.73	8.81	14.8
IC1	FQPTLLTLPR	57.95	14.89	12.92	3.35	1.5	11.6	4.5	5.93	12.67	9.26	10.43
IGHA1	TPLTATLSK	14.29	4.3	9.27	9.42	4.51	4.47	4.92	8.45	4.1	2.18	4.71
IGHG1	FNWYVDGVEVHNAK	31.19	23.52	19.66	5.88	5.55	10.01	23.55	5.64	11.74	19.5	15.62
IGHG3	NQVSLTCLVK	16.07	6.15	5.24	10.49	3.43	9.07	1.78	6.43	15.78	3.17	6.29
IGHM	QIQVSWLR	13.09	19.95	10.59	9.46	30.6	25.69	20.37	12.97	9.04	6.39	13.03
IGHM	YAATSQVLLPSK	18.97	23.08	19.26	26.01	22.1	33.49	13.36	26.26	8.22	19.3	20.7
ITIH1	LDAQASFLPK	23.32	38.66	49.14	12.31	14.62	9.75	24.86	41.07	9.13	31.93	24.09
ITIH2	FYNQVSTPLLR	38.4	17.58	11.92	16.57	31.21	10.51	14.78	18.94	13.78	27.77	17.07
ITIH2	IQPSGGTNINEALLR	50.15	16.52	28.04	12.97	11.02	25.87	6.88	2.48	15.02	4.09	14
ITIH4	ETLFSVMPGLK	27.42	38.73	26.78	35.03	30.2	14.19	12.4	7.36	40.81	27.2	27.31
KNG1	DFVQPPTK	16.11	10.8	5.59	6.37	9.19	7.78	1.16	1.16	16.45	5.09	7.08
LUM	SLEDLQLTHNK	18.79	14.2	5.85	7.02	10.08	12.75	23.1	6.82	8.85	8.4	9.46
PEDF	TVQAVLTVPK	31.97	18.03	13.74	35.17	34.01	1.82	20.7	31.29	8.74	33.3	25.99
PGRP2	TFTLLDPK	9.04	7.83	8.33	7.05	18.35	15.27	5.69	7.88	9.02	16.29	8.68
PHLD	NQVVIAAGR	28.41	33.15	21.07	21.49	12.6	44.83	21.42	7.04	9.56	12.27	21.25
PLMN	FVTWIEGVMR	40.32	4.96	25.31	16.41	9.95	9.34	5.99	9.99	3.36	6.06	9.64
SAMP	IVLGQEQDSYGGK	22.56	8.92	27.74	3.32	26.68	14.51	10.6	11.04	16	10.76	12.77
THRB	ELLESYIDGR	20.44	17.74	17.54	28.38	27.94	29.36	25.8	11.97	9.7	33.91	23.12
TRFE	EGYYGYTGAFR	71.36	58.04	8.56	20.3	34.85	59.65	13.85	69.49	29.68	39.98	37.41
TTHY	AADDTWEPFASGK	25.93	28.66	23.35	16.16	11.77	14.5	8.42	30.81	25.13	1.15	19.76
TTHY	VLDAVR	21.32	15.51	15.68	8.25	10.32	9.65	5.2	13.67	3.48	16.51	12
VTNC	DVWGIEGPIDAAFTR	121.93	25.41	5.52	19.26	20.48	20.58	35.07	10.9	10.74	7.16	19.87
VTNC	DWHGVPGQVDAAMAGR	15.64	16.08	19.94	9.37	13.89	25.87	24.32	30.13	8.48	20.8	18.01

Supplementary Table 3(b) Serum peptide transition sample to sample variability (geometric CV by healthy volunteer sample). S – healthy volunteer sample.

**Protein**	**Peptide**	**S1**	**S2**	**S3**	**S4**	**S5**	**S6**	**S7**	**S8**	**S9**	**S10**	**Median**
A1AT	SVLGQLGITK	48.67	10.96	10.12	1.63	1.87	20.16	9.38	7.67	11.42	3.79	9.75
A1BG	ATWSGAVLAGR	16.99	5.99	3.5	4.37	5.25	17.7	3.43	17.89	16.05	9.83	7.91
A1BG	CLAPLEGAR	10.43	23.26	8.05	18.42	17.32	9.43	24.93	11.39	16.34	11.13	13.87
A1BG	SGLSTGWTQLSK	18.81	38.2	9.35	14.4	69.58	32.11	23.66	28.52	4.59	12.6	21.23
A2AP	DFLQSLK	28.58	10.7	12.14	3.3	1.56	0.71	11.93	16.11	7.89	8.22	9.46
A2MG	NEDSLVFVQTDK	14.55	13.29	10.11	19.25	8.3	14.44	21.83	19.73	12.78	9.54	13.86
AACT	ADLSGITGAR	22.51	2.26	12.15	5.34	10.46	14.82	15.89	9.57	9.58	7.65	10.02
AACT	EIGELYLPK	24.58	18.18	20.91	5.14	14.52	14.19	6.76	5.42	10.44	17.63	14.36
ALBU	AAFTECCQAADK	25.84	24.89	15.37	12.47	9.01	17.59	3.28	6.79	2.66	2.55	10.74
ALBU	ETYGEMADCCAK	21.35	8.91	16.44	13.62	3.91	31.06	28.94	12.43	13.63	45.48	15.04
AMBP	TVAACNLPIVR	22.01	6.04	23.61	5.69	22.28	16.04	21.61	8.66	12.57	25.25	18.82
ANGT	ALQDQLVLVAAK	51.21	29.9	1	30.54	6.19	17.32	8.24	21.65	8.95	8.22	13.13
ANGT	SLDFTELDVAAEK	48.95	14.01	10.79	15.74	8.45	15.08	5.18	17.68	8.43	10.7	12.4
ANT3	LPGIVAEGR	15.91	15.08	26.76	14.8	28.01	29.08	20.74	7.28	19.65	18.15	18.9
APOA1	ATEHLSTLSEK	22.72	24.61	8.16	21.74	23.59	3.17	17.94	21.97	13.67	11.48	19.84
APOA2	SPELQAEAK	28.63	12.83	15	9.58	7.8	9.09	11.47	13.41	7.45	22.7	12.15
APOA4	ALVQQMEQLR	42.03	34.92	50.82	8.91	61.26	22.74	14.96	24.71	39.56	40.09	37.24
APOA4	ISASAEELR	22.49	18.26	3.94	18.22	13.83	2.56	11.71	2	5.43	6.17	8.94
APOC1	EFGNTLEDK	24.54	15.6	6.72	21.68	19.37	5.81	14.88	11.01	10.13	4.12	12.95
APOC1	EWFSETFQK	29.6	31.56	14.29	29.89	9.91	10.99	14.12	15.76	40.04	6.71	15.02
APOC2	TAAQNLYEK	24.79	5.36	4.49	30.25	19.68	29.6	4.25	9.29	25.77	16.28	17.98
APOC3	GWVTDGFSSLK	36.98	13.7	18.61	10.19	14.15	8.03	23.9	6.82	4.59	5.06	11.95
APOC4	AWFLESK	10.38	17.4	22.57	40.25	20.44	14.9	11.41	11.69	2.92	11.53	13.29
APOD	VLNQELR	18.78	19.77	12.69	5.4	15.09	4.72	4.15	9.48	5.89	8.68	9.08

APOE	AATVGSLAGQPLQER	30.17	17.04	16.65	15.66	46.7	11.48	3.71	16.05	12.07	6.82	15.86
APOE	SELEEQLTPVAEETR	53.48	17.27	14.26	8.99	36.4	18.9	18.97	27.2	31.12	38.22	23.09
APOH	VSFFCK	16.49	17.79	21.43	8.12	16.22	7.06	23.87	3.27	28.15	24.85	17.14
APOL1	VTEPISAESGEQVER	30.62	40.82	29.56	4.37	14.73	44.08	24.68	12.94	17.39	38.25	27.12
APOM	AFLLTPR	13.51	8.23	3.26	10.35	7.58	2.97	7.18	3.51	13.56	6	7.38
APOM	SLTSCLDSK	27.82	48.84	26.64	13.47	11.63	19.28	12.58	17.95	6.37	23.34	18.62
C1R	YTTEIIK	30.22	6.35	25.08	13.12	13.89	16.2	10.05	11.61	12.76	7.41	12.94
C1S	LLEVPEGR	13.65	9.44	15.85	17.41	14.77	16.14	1.6	3.98	15.5	13.08	14.21
C1S	TNFDNDIALVR	33.14	10.33	12.44	23.9	8.85	9.92	20.63	8.83	13.62	14.61	13.03
C4BPA	EDVYVVGTVLR	39.55	16.53	5.05	8.83	10.2	9.59	8.19	14.91	9.63	11.49	9.91
C4BPA	YTCLPGYVR	26.94	15.05	20.13	12.44	9.76	18.2	10.4	3.09	24.39	13.22	14.14
CBG	GTWTQPFDLASTR	50.88	12.01	16.36	4.98	21.58	13.33	8.92	13.27	33.62	13.37	13.35
CERU	EVGPTNADPVCLAK	21.34	21.67	23.82	6.16	14.8	37.57	24.59	14.37	22.33	4.87	21.51
CFAB	DISEVVTPR	20.03	19.8	13.1	31.38	7.57	26.5	9.88	14.07	26.04	3.26	16.94
CFAB	DLLYIGK	21.9	1.41	6.44	2.2	6.12	2.4	14.26	2.29	9.29	4.51	5.31
CFAB	EELLPAQDIK	9.89	6.43	20.51	23.1	15.59	8.84	4.4	9.99	12.72	2.28	9.94
CFAB	YGLVTYATYPK	38.69	36.93	27.01	29.52	14.75	17.99	6.09	37.91	31.18	9.65	28.27
CLUS	IDSLLENDR	23.29	24.57	15.35	8.2	21.39	2.07	6.5	19.94	34.53	12.19	17.64
CO4A	DFALLSLQVPLK	42.6	14.15	21.81	21.18	17.84	7.16	3.87	17.9	5.74	22.33	17.87
CO4A	ITQVLHFTK	14.33	12.84	15.33	15.01	16.79	17.21	12.2	9.76	14.46	1.72	14.39
CO8A	MESLGITSR	28.91	3.21	34.73	14.79	4.54	20.8	1.1	12.3	12.14	26.02	13.54
CO9	LSPIYNLVPVK	54.77	8.06	13.54	8.85	19.23	9.7	6.43	3.66	5.3	15.12	9.27
CO9	VVEESELAR	14.76	7.63	2.37	36.4	19.1	3.38	6.47	28.99	11.27	7.97	9.62
FA12	VVGGLVALR	12.65	5.39	14.01	8.5	7.74	18.71	8.06	4.24	17.32	17.97	10.57
FETUA	FSVVYAK	12.27	10.95	2.24	16.39	9.11	6.74	7.04	2.91	13.92	16.61	10.03
FETUA	HTLNQIDEVK	12.16	8.77	2.88	7.19	6.07	14.97	5.06	8.11	8.8	5.54	7.65
GELS	AGALNSNDAFVLK	9.2	4.66	13.35	20.69	20.94	28.72	20.89	14.05	11.87	41.44	17.37
GELS	SEDCFILDHGK	23.98	10.6	12.1	20.31	5.7	17.56	7.63	13.38	14.2	11.65	12.74
HEMO	NFPSPVDAAFR	36.14	7.68	6	3.97	3.53	10.17	6.97	4.96	3.78	1.62	5.48
HEMO	VDGALCMEK	29.46	29.61	23.25	6.39	15.4	17.15	16.99	21.56	32.04	27.88	22.41
HEP2	FAFNLYR	25.22	11.16	15.09	3.46	13.85	15.04	2.44	16.12	12.29	8.19	13.07

HEP2	IAIDLFK	18	9.55	9.31	2.94	7.47	3.59	18.12	13.52	9.58	15.31	9.57
HPT	VGYVSGWGR	22.41	10.64	11.12	6.48	11.9	12.93	15.47	5.83	12.66	6.07	11.51
HPT	VTSIQDWVQK	25.05	19.05	4.08	15.87	7.74	1.06	21.13	21.76	13.73	8.81	14.8
IC1	FQPTLLTLPR	57.95	14.89	12.92	3.35	1.5	11.6	4.5	5.93	12.67	9.26	10.43
IGHA1	TPLTATLSK	14.29	4.3	9.27	9.42	4.51	4.47	4.92	8.45	4.1	2.18	4.71
IGHG1	FNWYVDGVEVHNAK	31.19	23.52	19.66	5.88	5.55	10.01	23.55	5.64	11.74	19.5	15.62
IGHG3	NQVSLTCLVK	16.07	6.15	5.24	10.49	3.43	9.07	1.78	6.43	15.78	3.17	6.29
IGHM	QIQVSWLR	13.09	19.95	10.59	9.46	30.6	25.69	20.37	12.97	9.04	6.39	13.03
IGHM	YAATSQVLLPSK	18.97	23.08	19.26	26.01	22.1	33.49	13.36	26.26	8.22	19.3	20.7
ITIH1	LDAQASFLPK	23.32	38.66	49.14	12.31	14.62	9.75	24.86	41.07	9.13	31.93	24.09
ITIH2	FYNQVSTPLLR	38.4	17.58	11.92	16.57	31.21	10.51	14.78	18.94	13.78	27.77	17.07
ITIH2	IQPSGGTNINEALLR	50.15	16.52	28.04	12.97	11.02	25.87	6.88	2.48	15.02	4.09	14
ITIH4	ETLFSVMPGLK	27.42	38.73	26.78	35.03	30.2	14.19	12.4	7.36	40.81	27.2	27.31
KNG1	DFVQPPTK	16.11	10.8	5.59	6.37	9.19	7.78	1.16	1.16	16.45	5.09	7.08
LUM	SLEDLQLTHNK	18.79	14.2	5.85	7.02	10.08	12.75	23.1	6.82	8.85	8.4	9.46
PEDF	TVQAVLTVPK	31.97	18.03	13.74	35.17	34.01	1.82	20.7	31.29	8.74	33.3	25.99
PGRP2	TFTLLDPK	9.04	7.83	8.33	7.05	18.35	15.27	5.69	7.88	9.02	16.29	8.68
PHLD	NQVVIAAGR	28.41	33.15	21.07	21.49	12.6	44.83	21.42	7.04	9.56	12.27	21.25
PLMN	FVTWIEGVMR	40.32	4.96	25.31	16.41	9.95	9.34	5.99	9.99	3.36	6.06	9.64
SAMP	IVLGQEQDSYGGK	22.56	8.92	27.74	3.32	26.68	14.51	10.6	11.04	16	10.76	12.77
THRB	ELLESYIDGR	20.44	17.74	17.54	28.38	27.94	29.36	25.8	11.97	9.7	33.91	23.12
TRFE	EGYYGYTGAFR	71.36	58.04	8.56	20.3	34.85	59.65	13.85	69.49	29.68	39.98	37.41
TTHY	AADDTWEPFASGK	25.93	28.66	23.35	16.16	11.77	14.5	8.42	30.81	25.13	1.15	19.76
TTHY	VLDAVR	21.32	15.51	15.68	8.25	10.32	9.65	5.2	13.67	3.48	16.51	12
VTNC	DVWGIEGPIDAAFTR	121.93	25.41	5.52	19.26	20.48	20.58	35.07	10.9	10.74	7.16	19.87
VTNC	DWHGVPGQVDAAMAGR	15.64	16.08	19.94	9.37	13.89	25.87	24.32	30.13	8.48	20.8	18.01
